# Sustainable treatment of banana leaves for phytosanitary applications: impact, spreading, and impregnation of mineral oil

**DOI:** 10.1002/ps.70660

**Published:** 2026-02-18

**Authors:** Abdallah Alayan, Stéphane Boyer, Jean‐Luc Verdeil, Fréderic Gatineau, Patrick Hermet, Jean‐Louis Bantignies, Christian Ligoure

**Affiliations:** ^1^ Laboratoire Charles Coulomb (L2C) Université de Montpellier, CNRS Montpellier France; ^2^ CIRAD UMR AGAP Institut, PHIV Montpellier France; ^3^ UMR AGAP Institut‐PHIV Université de Montpellier, CIRAD, INRAE, Institut Agro Montpellier France; ^4^ Institut Charles Gerhardt Montpellier (ICGM) Université de Montpellier, CNRS Montpellier France

**Keywords:** phytosanitary spray, mineral oil, droplet dynamics, leaf penetration, banana disease, μFTIR, infrared, impregnation, diffusion kinetics

## Abstract

**BACKGROUND:**

Efficient application of phytosanitary sprays is essential for sustainable control of foliar fungal diseases such as Black Sigatoka in banana crops. Mineral oils are commonly used for their fungistatic properties, yet their modes‐of‐action, particularly their interactions with leaf tissues, remain poorly understood. This study aims to elucidate the physical behavior of mineral oil droplets on banana leaves and their subsequent diffusion into internal tissues.

**RESULTS:**

High‐speed imaging shows that mineral oil droplets reach their maximum spread without retraction and exhibit only low splashing at high impact velocities. Spray coverage is strongly anisotropic and increases over time following a power‐law scaling (*t* ^*a*^ with *α* = 0.21 ± 0.02), in agreement with Tanner's law. Micro‐infrared spectroscopy reveals that mineral oil penetrates the leaf, with preferential accumulation in the palisade parenchyma. Diffusion into internal leaf tissues, specifically the palisade parenchyma, follows Fick's law after a latency of ≈3.6 h, with an effective diffusion coefficient of (1.2 ± 0.8) × 10^−8^ cm^2^ s^−1^.

**CONCLUSION:**

This study provides the first direct evidence that mineral oils not only protect leaf surfaces, but also diffuse into internal tissues targeted by fungal pathogens. These findings offer a mechanistic basis for field practices and support the development of more effective and sustainable foliar spray formulations. © 2026 The Author(s). *Pest Management Science* published by John Wiley & Sons Ltd on behalf of Society of Chemical Industry.

## Graphical abstract

Mineral oil droplets spread anisotropically, penetrate banana leaf tissues, and diffuse into the palisade parenchyma, revealing a delayed, Fickian mechanism that supports improved phytosanitary spray strategies.

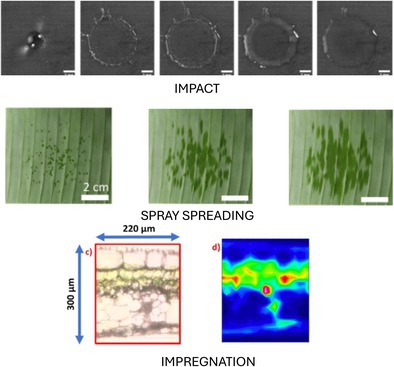

## INTRODUCTION

Banana (*Musa* spp.) is a key staple and commercial crop in tropical and subtropical regions. However, its production is severely threatened by foliar fungal diseases, the most damaging of which is black Sigatoka, caused by the pathogen *Pseudocercospora fijiensis*. This disease significantly reduces both yield and fruit quality, shortens the productive lifespan of banana plants and increases vulnerability to secondary infections. Traditional disease management relies heavily on frequent fungicide applications^1^, ^2^, ^3^ In addition to their environmental footprint, intensive fungicide‐based strategies raise concerns related to resistance development, regulatory restrictions, and compatibility with organic and integrated pest management (IPM) systems, motivating the search for alternative or complementary solutions.

In response to these challenges, paraffinic mineral oils have gained renewed attention in crop protection. These oils exhibit fungistatic properties, including the inhibition of spore germination and suppression of symptom expression during early infection stages.^4^ Their compatibility with integrated pest management and organic systems makes them promising alternatives to synthetic fungicides. Studies have demonstrated that paraffinic oils, such as ©Banole, can improve disease control either alone or in combination with fungicides^5^, ^6^

Beyond their well‐documented fungistatic effects, mineral oils may interact with leaves through multiple, nonexclusive mechanisms, including the formation of a physical barrier at the leaf surface, direct penetration into internal tissues targeted during early infection stages, and potential modulation of local plant defense responses. However, experimental evidence supporting these mechanisms remains limited.

However, most existing studies have focused on mineral oils within complex formulations or as adjuvants rather than as primary active substances. The standalone role of mineral oil—the dominant ingredient in such formulations—remains underexplored. Yet understanding its individual behavior is essential: not only does it largely determine the formulation's physical and chemical properties, but it also may provide protective effects as a biologically active agent, reducing the need for toxic fungicides.

The dynamic impact of droplets and spray spreading on leaf surface is a key factor in the fungal treatment of leaves. It is influenced by interactions between numerous controllable and uncontrollable variables^7^ including surface tension, viscosity of the spray solution, droplet size and velocity, ejection speed at the nozzle outlet, droplet trajectory before impact, surface topology of the leaves, temperature, relative humidity (RH) and wind speed.

Jia and Zhu^8^ studied the interaction of water droplets with soybean leaves regarding their impact, rebound, retention and spreading. They showed that droplet coverage on leaves increases with droplet size and impact velocity. Dong *et al*.^9^ demonstrated that the presence of surfactants inhibits droplet rebound on the leaf surface. Gilet and Bourouiba^10^ explored the dynamics of rain‐induced pathogen transmission in foliar diseases, identifying two main ejection mechanisms: crescent‐moon and inertial detachment. These mechanisms depend on leaf characteristics and influence pathogen spread differently. Findings suggest that leaf compliance affects the range of contaminated droplet ejection and, consequently, disease transmission. Bassette and Bussière^11^ quantifies how raindrop size, kinetic energy and leaf inclination govern splash, storage and dripping on banana foliage, providing empirical relationships that enable realistic modelling of rainfall interception and subsequent water flows to better manage erosion, agrochemical leaching and disease risks in banana plantations. Jiang *et al*.^12^ investigated the asymmetric spreading of pesticide droplets on inclined banana leaves, finding that increased leaf inclination and droplet impact velocity heighten the asymmetry factor. Theoretical models were developed to describe tangential and lateral spreading diameters, as well as the slip distance on inclined leaves. As impact velocity increases, energy dissipation occurs owing to interactions with the leaf's non‐smooth surface, reducing droplet rebound. The study of droplet impact on leaves is therefore highly relevant for characterizing interactions between leaves and phytosanitary sprays.

A second key factor for fungal treatments is the ability of droplets to penetrate the heart of the leaf. The penetration of mineral oil into the cuticle was reported by Laville *et al*.^13^ using optical microscopy. For examining the impregnation of organic species, micro‐Fourier transform infrared spectroscopy (μFTIR) is a powerful technique that probes molecular fingerprints based on dynamic dipolar variations and provides chemical maps through the thickness of leaves at the microstructural scale.^14^, ^15^, ^16^, ^17^ The mechanism of water retention in mature leaves of *Cryptomeria japonica* D. was studied using μFTIR as a function of tree height^16^ showing that water retained in tall *C. japonica*.

In this article, we study, for the first time to the best of our knowledge, the full sequence of interactions between mineral oil and banana leaves during phytosanitary treatments, considering the successive processes of droplet impact, anisotropic spreading and internal tissue impregnation. Our experimental approach combines high‐speed imaging, quantitative image analysis, and μFTIR to investigate both surface‐level dynamics and sub‐surface diffusion under conditions mimicking real‐world field applications. This integrated physicochemical framework enables us to (i) characterize the impact behavior of oil droplets on a biologically relevant, multiscale, and anisotropic leaf surface, (ii) quantify the time‐resolved spreading of spray droplets and, (iii) provide the first direct and quantitative evidence of mineral oil diffusion into the internal leaf tissues—specifically into the palisade parenchyma.

These original findings offer new insights into the physical and chemical mechanisms underlying the efficacy of mineral oils against foliar pathogens such as *Mycrosphaerellafijiensis*. By bridging microscale physical processes and tissue‐level transport, this work lays the foundation for the rational design of next‐generation phytosanitary formulations better suited to the structural and functional complexity of plant surfaces. The methodology developed here is broadly transferable and may contribute to more effective and sustainable crop protection strategies across diverse agricultural systems.

## MATERIALS AND METHODS

### Mineral oil

The oil used in this study is a commercial mineral oil known as ©MaxPar BSO 75. This high‐purity mineral spraying oil is commonly used as a carrier fluid for phytosanitary products in agriculture. ©MaxPar BSO 75 serves as a carrier fluid for fungicides aimed at controlling both Yellow Sigatoka and Black Sigatoka diseases. This oil also is used in its pure form for organic treatments of these diseases. Its density is 0.82 g mL^−1^, its molar mass is 314 g mL^−1^, its viscosity, measured with an MCR302 rheometer (Anton Paar, Graz, Austria) in Couette geometry, is 14 ± 1 mPa·s, and its surface tension, measured using a pendant drop tensiometer (PATIM, Sinterface Technologies, Berlin, UK), is 23 ± 1 mN m^−1^.

### Banana leaf

The banana leaves used in this study belong to the Cavendish Musa variety (*Musa accuminata*).

For impregnation studies and drop impact experiments, we used a young, fully expanded banana leaf [Fig. 4(a)] corresponding to the first leaf unfolded after the cigar stage.

The cigar corresponds to a banana leaf tightly curl when young; it unfurls as it grows or rehydrates. Thus, the leaf unfurls when a second rolled‐up leaf appears as the banana tree grows.

#### Surface wettability

Like most plants, the surface of a banana leaf is hydrophobic.^18^ When a water droplet is deposited on the surface of a banana leaf, which is hydrophobic, it forms a contact angle of ≈117 ± 4°. By contrast, an oil droplet spreads completely, exhibiting total wetting with a zero‐contact angle. In all of the experiments presented in this study, only the upper surface (adaxial) of the leaf was investigated, as it is the one directly exposed to droplets during spraying.

#### Surface roughness

**Figure 1:**
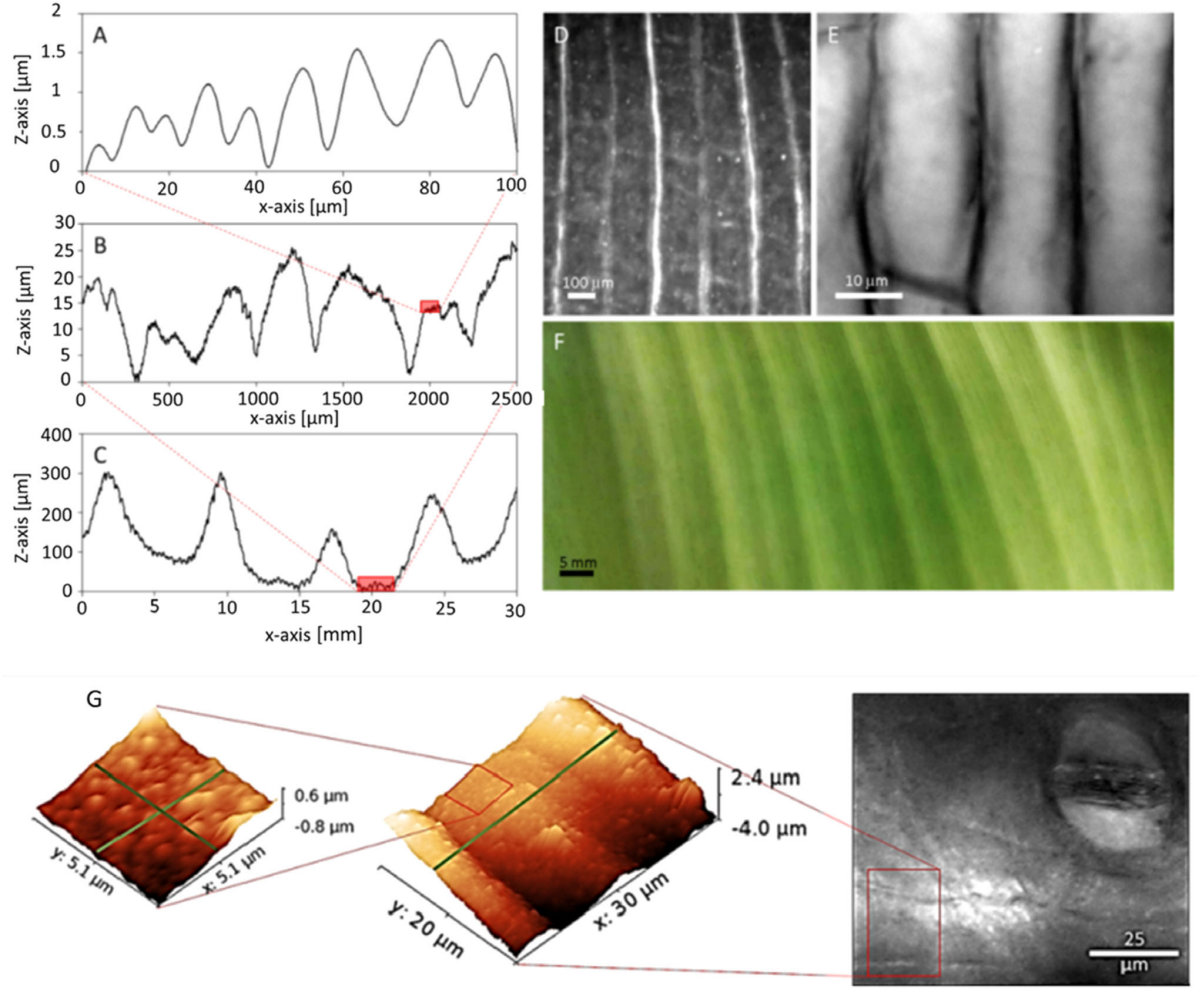
Typical abaxial surface roughness structures of a banana leaf at various scales, obtained by profilometry (measurement direction perpendicular to grooves) (a–c), accompanied by corresponding surface images obtained via microscopy (d–f), and isotropic roughness of the banana leaf identified by AFM (g).

We characterize the roughness of banana leaves (abaxial surface) using profilometry at millimetric and micrometric scales, and atomic force microscopy (AFM) at micrometric and nanometric scales (Fig. 1). The results demonstrate that the banana leaf surface is multitextured, exhibiting anisotropic roughness with a periodic structure at different scales, each associated with characteristic elevation amplitudes:

- A first roughness pattern at a large scale, perpendicular to the midrib, with peak‐to‐peak distances of 7 ± 1 mm and peak heights of 250 ± 80 μm [Fig. 1(C), (F)].
- A second roughness pattern, also perpendicular to the midrib, with peak‐to‐peak distances of 200 ± 50 μm and peak heights of 15 ± 5 μm [Fig. 1(B), (D)]
- A third roughness pattern, again perpendicular to the midrib, with peak‐to‐peak distances of 20 ± 5 μm and peak heights of 2 ± 0.5 μm [Fig. 1(A), (E)].
- A fourth, isotropic roughness pattern, identified by AFM, with peak‐to‐peak distances of 1 ± 0.5 μm and peak heights of 0.2 ± 0.05 μm [Fig. 1(G)].

#### Cross‐section

**Figure 2:**
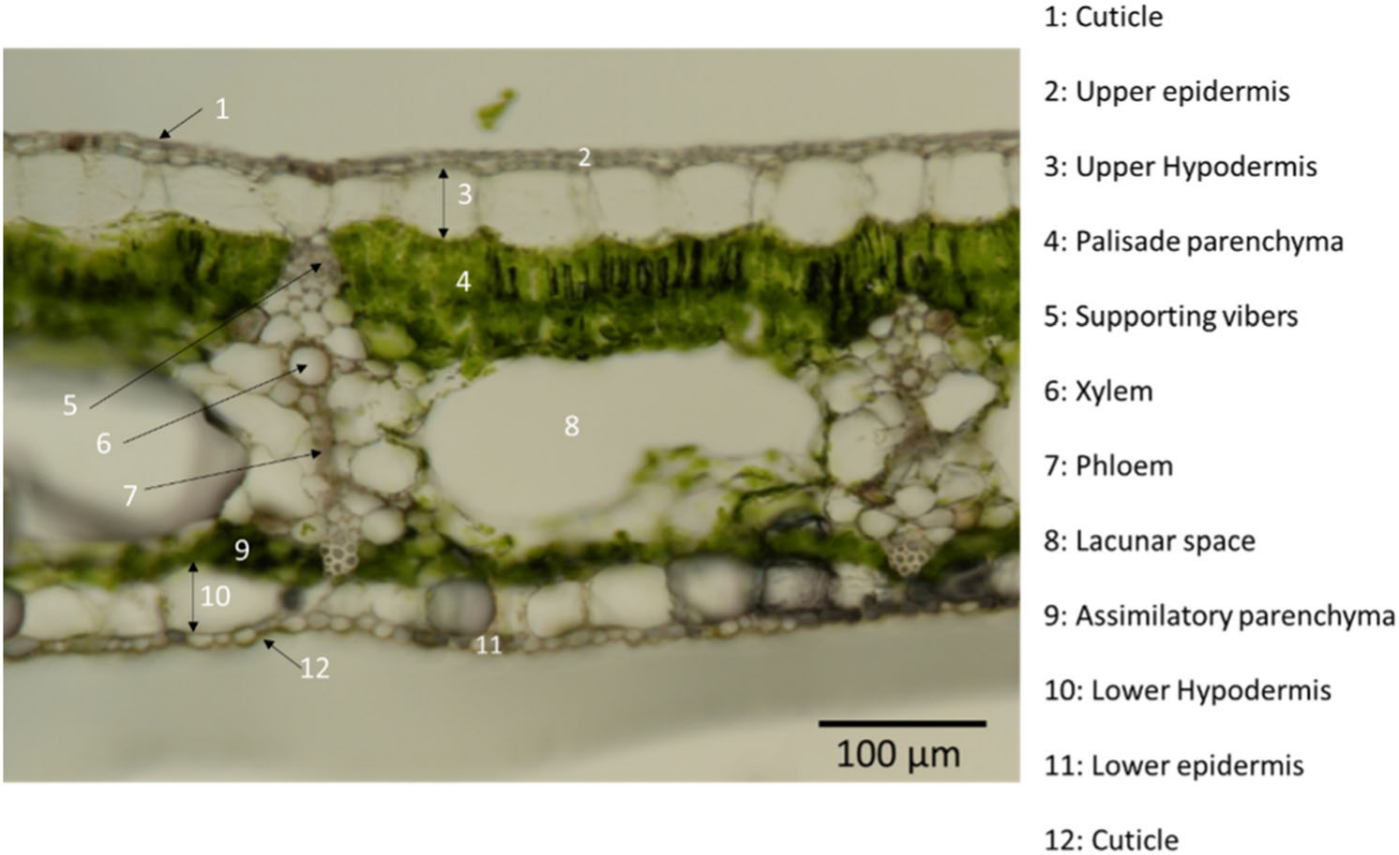
Cross‐section of a banana leaf lamina, perpendicular to the secondary veins, with a thickness of 40 μm, prepared using a vibratome observed by Leica optical microscopy ×50.

For the impregnation studies, we opted for thin cross‐sections of banana leaves, in the middle of the leaf, to enable analysis through μFTIR in transmission mode. These sections were prepared with a thickness of *l* = 40 μm, optimized for IR transmission, using a vibratome (Microm HM 650V; Thermo Fisher Scientific, Waltham, MA, USA). Before sectioning, the banana leaf samples were encapsulated embedded in a 4% (v/v) agarose gel. The structure of the banana leaf consists of several layers of tissues, each fulfilling different functions for the plant (see Fig. 2).

### Drop impact

**Figure 3:**
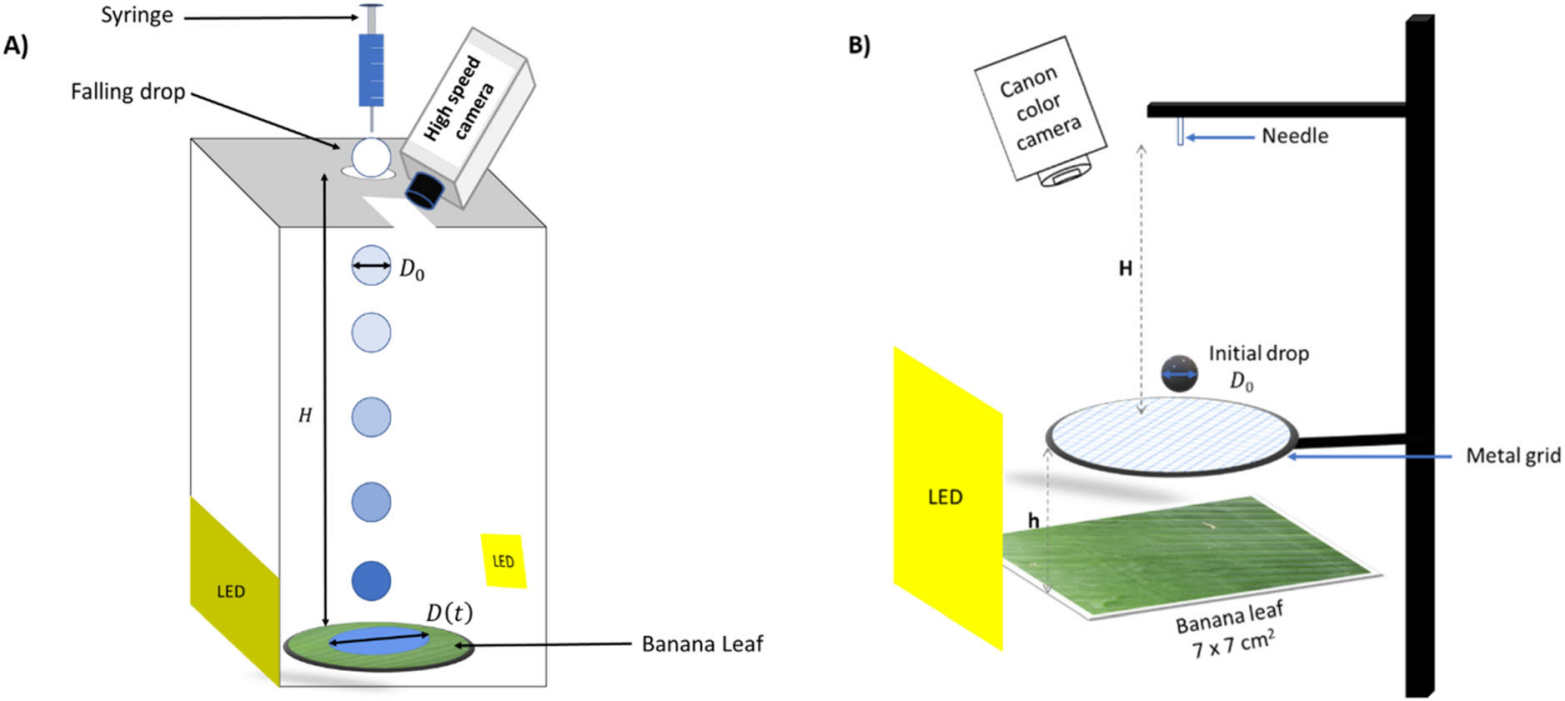
Schematic illustrations of the experimental setup for (a) drop impact experiments and (b) spray spreading experiments.

Droplets of the mineral oil were delivered using a syringe pump operating at a constant volumetric flow rate of 1 mL min^−1^ and connected, via a flexible Teflon tube, to a needle (with internal diameter 2 mm) positioned vertically above the impact zone. The leaf sample was selected and affixed onto a flat substrate using adhesive tape [see experimental setup in Fig. 3(A)]. The impacted droplets are spherical, with an initial diameter *D*_0_ and a projected surface area before impact given by $\pi D_{0}^{2}/4$. In our experiments, *D*_0_ varied between 3 and 3.2 mm. The release height determines the droplet's impact velocity, which follows the free‐fall law with zero initial velocity, neglecting air friction:

$$v_{0}=\sqrt{2\mathrm{gH}},$$

where *H* is the height from which the droplet is released and *g* is the gravitational acceleration. Vernay^19^ has shown that this approximation is highly accurate for impact velocities <7 m s^−1^. The impact events were recorded using a high‐speed camera (Phantom VEO 1310, Vision Resarch, Inc., Wayne, NJ, USA), operating at 10000 fps with a resolution of 1280 800 pixels. The camera was angled at ≈10° relative to the vertical axisplane. Proper illumination for acquisition was provided by high‐intensity backlighting: Phlox HSC^2^ and PHLOX ‐ LEDW ‐ BL ‐ 50 × 50. The temporal evolution of the droplet's diameter was measured through image analysis using imagej software. This enabled the detection of the droplet contour and the measurement of its area *A*(*t*), the projected area of the droplet's surface at time *t*. The diameter of the droplet is then determined as $D\left(t\right)=\sqrt{4A\left(t\right)/\pi }$. The results were obtained by averaging, for each sample, the temporal evolution of the impact parameter $\beta \left(t\right)=\frac{D\left(t\right)}{D_{0}}$ from three independent experiments; error bars correspond to the standard deviation.

### Spray spreading

In order to generate the model spray, a hydrophilic circular metal grid with circular diameter much larger than the drop diameter, with a wire diameter of 0.25 mm and a 0.23‐mm mesh size ‐(McMaster Carr, Elmhurst, IL, USA) was used. A 3.3‐mm‐diameter drop was released onto the grid from a height *H* = 30 cm, and a 7 × 7 cm^2^ banana‐leaf sample was placed beneath the grid at a constant height *h* of 4 cm [Fig. 3(B)]. This grid was previously employed by Park *et al*.^20^ to study droplet rebound on plant surfaces. A EOS 4000D (Canon, Tokyo, USA) color camera was positioned above the sheet at a 10° angle to capture successive images of the spreading of the formulation on the banana leaf. A Phlox HSC lighting system was used. To monitor this spreading on the leaf, the grid was removed so as not to obstruct the camera's view. The estimated time between the drop impact and the removal of the grid was 5 s.

### Impregnation

#### Experimental protocol for thick film impregnation

**Figure 4:**
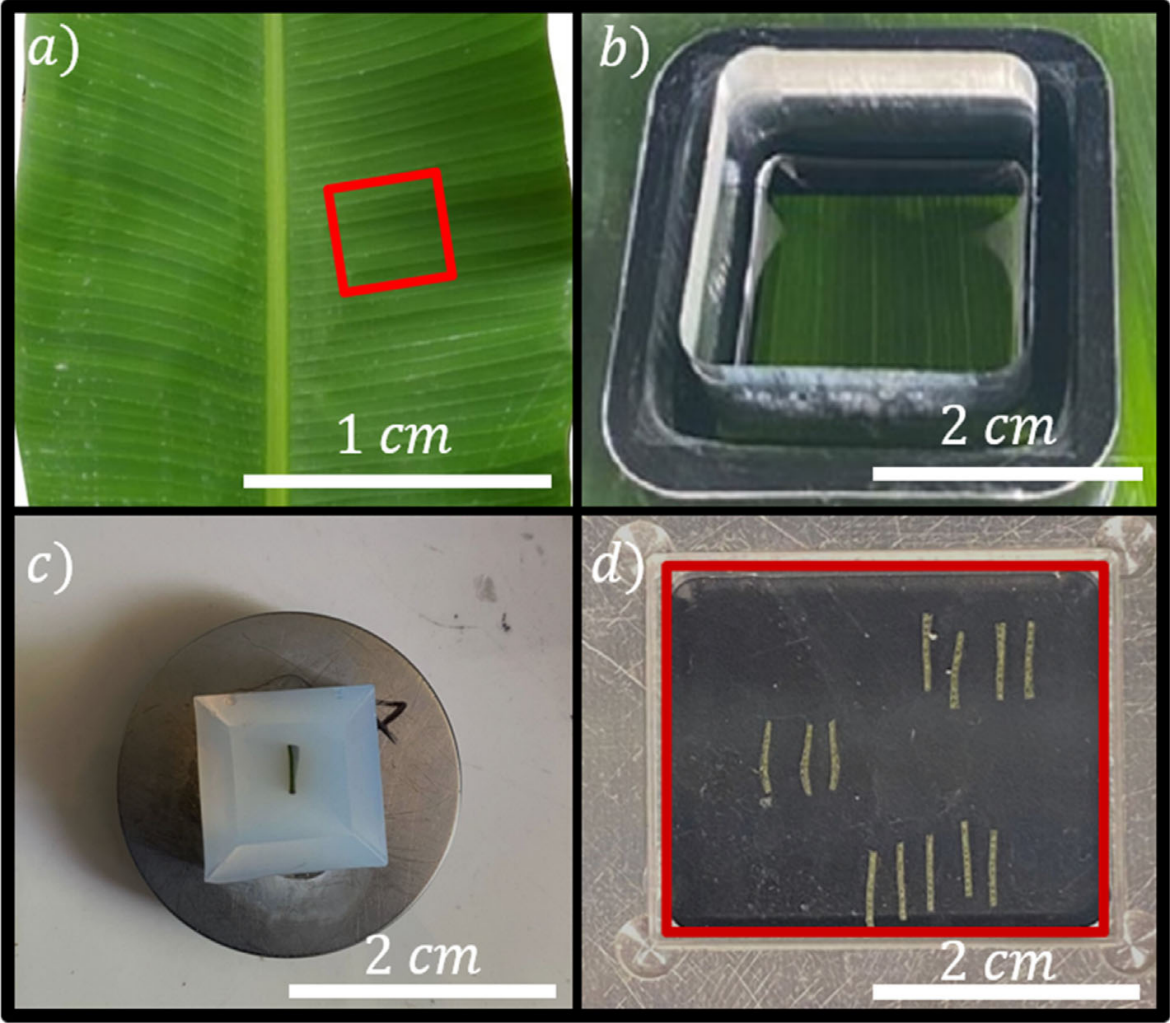
Experimental protocol steps for impregnation studies using an infinite film: (a) selection of the leaf portion, (b) application of oil to the upper surface of the banana leaf, (c) transfer of the banana leaf piece into agarose before cutting, and (d) final cross‐sections of the banana leaf.

In order to ensure reproducible oil impregnation conditions, a portion from the middle of the leaf blade was used [Fig. 4(a)]. Impregnation was carried out by placing an oil reservoir on the top side of the previously cut sheet [Fig. 4(b)]. The thickness of the oil film in the reservoir wass chosen to be 2 cm, much greater than the capillary length of the oil (1.7 mm) preventing any side effects at the interface between the oil and the leaf. The weight of the parallelepiped reservoir prevents leaks at the interface between its walls and the leaf surface, ensuring reproducible impregnation conditions.

After impregnation, the surface of the banana leaf was rinsed with water until the surface of the leaf no longer showed any traces of the impregnating agent. After rinsing, the sample was gently air‐dried to remove any residual surface water before embedding into agarose (see Section 2.2.3 for details), ensuring that infrared (IR) measurements were not affected by surface artifacts. Once the agarose had solidified, the polymer block containing the leaf was attached to a vibratome support [Fig. 4(c)]. Subsequently, 40‐μm cross‐sections were made. The thickness was chosen based on preliminary studies to prevent saturation of the IR signal in transmission mode and to optimize the signal‐to‐noise ratio. The cross‐sections were then transferred onto a CaF2 substrate, which is transparent in the middle IR domain (4000–800 cm^−1^) before observation [Fig. 4(d)].

#### Experimental protocol for spray impregnation

We followed the experimental protocol described in Section 2.4. After the impregnation of the oil spray, the sample was left to rest for 24 h under ambient conditions before preparing the leaf cross‐sections for IR investigation.

#### Micro‐infrared spectroscopy

Infrared experiments were carried out in the middle infrared (MIR) domain using an Invenio FTIR spectrometer (Bruker, Billerica, MA, USA) in transmission mode, where 128 scans are co‐added with a resolution of 4 cm^−1^. This spectrometer was coupled with a Bruker Hyperion 2000 microscope, allowing μFTIR studies that combine optical and chemical imaging of cross‐histological cuts of the banana leaf. We used Cassegrain ×20 objectives with a numerical aperture (NA) of 0.6, a GLOBAR‐type blackbody source, a KBr beamsplitter and a nitrogen‐cooled photovoltaic MCT detector (mercury‐cadmium‐telluride). The IR beam size resolution, limited by the brightness of the internal source, was 20 × 20 μm^2^.

Chemical maps were obtained by measuring the absorbance of the stretching CH modes *ν*(CH), specific to the oil fingerprint, as a function of the position within the thickness of the leaf. The IR map represents, for each pixel, the integrated absorbance *A*(*ν*(CH)) of the *ν*(CH) band between 3000 and 2800 cm^−1^, after subtraction of a local linear background in the wavenumber domain. All maps use the same intensity scale, ensuring consistency.

**Figure 5:**
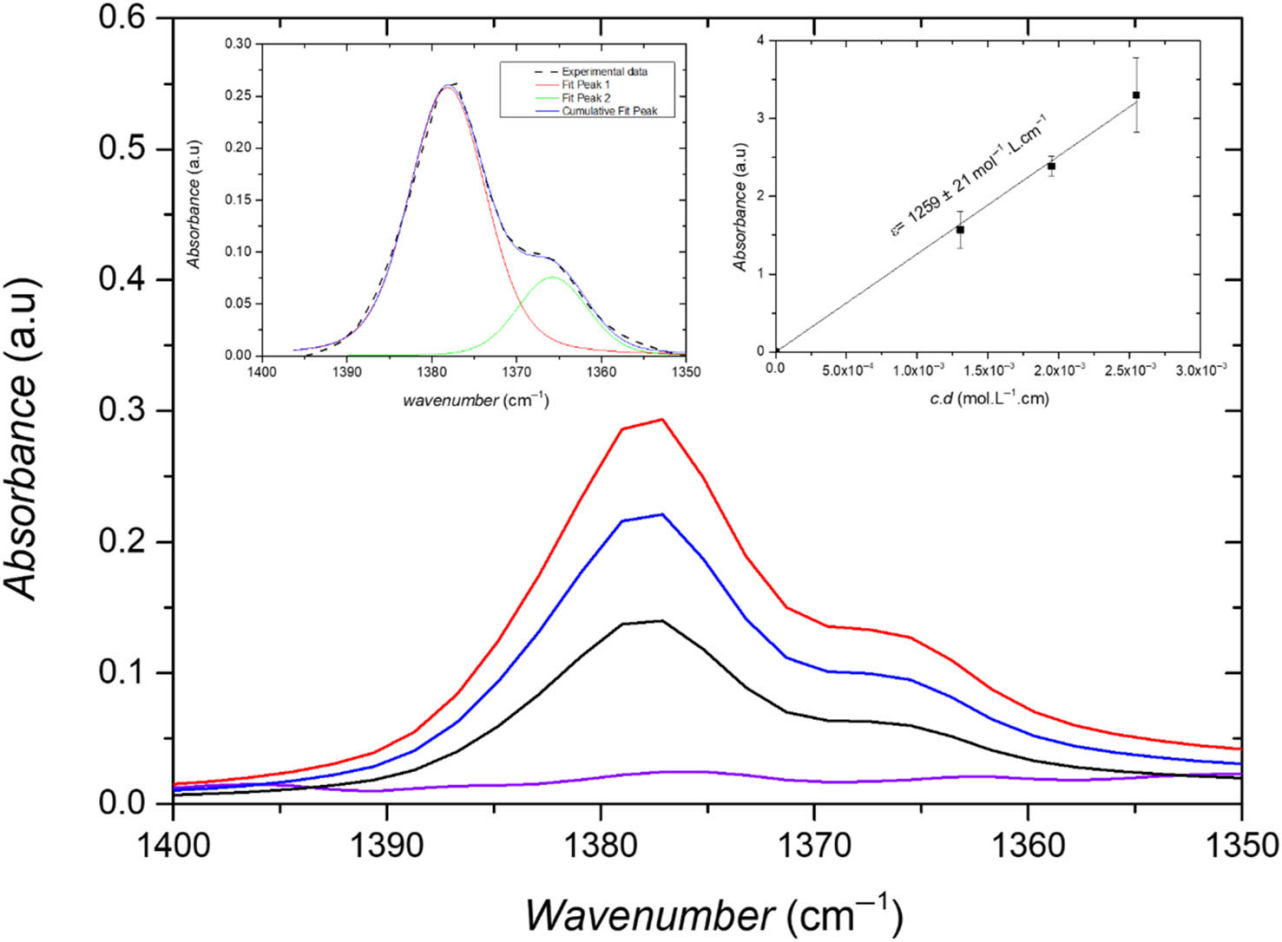
Infrared absorbance spectra of cyclohexane (purple) and different mineral oil volume fractions in cyclohexane (C1: black, C2: blue, C3: red), corresponding respectively to the volume fractions (13.1, 19.5, 25.5 mol L^−1^) in the wavenumber range 1400–1350 cm^−1^. Right inset: Absorbance calibration curve as a function of the concentration of mineral oil in cyclohexane (symbols: experimental data, plain line: linear fit), leading to *ε*_*ν*(CH)_ = 1259 ± 21 mol^−1^·L·cm. Left inset: Fitting of the experimental data for concentration C1 using two Voigt functions after subtraction of a linear background between 1400 and 1350 cm^−1^ superimposed on the experimental data.

In order to quantify the concentration of impregnated oil inside the leaf from IR spectroscopy, Beer–Lambert's law was applied. The determination of a calibration curve showing the linear dependence of *A*(ν(CH)) on the concentration was required. Different concentrations of oil were prepared by diluting the mineral oil in cyclohexane as a solvent (see Fig. 5). Cyclohexane was chosen as a solvent because it is nonpolar, fully miscible with mineral oil. Because the *ν*(CH) bands of cyclohexane and oil overlap in the 2800–3000 cm^−1^ range, it is not possible to perform the calibration in the domain. The calibration curve was calculated using the integrated absorbance *A*(*δ*(CH)) of the alkyl deformation band *δ*(CH) in the 1400–1350 cm^−1^ range where no band of the cyclohexane was exhibited. Two bands assigned to *δ*(CH) of oil centered at 1378 and 1365 cm^−1^ are shown (Fig. 5). The integrated absorbances *A*(δ(CH)) of the two bands was firstly carried out by fitting using Voigt functions in the 1400–1350 cm^−1^ range after linear background subtraction between 1400 and 1350 cm^−1^ (see Fig. 5, left inset). The *A*(*δ*(CH)) of the main alkyl deformation band *δ*(CH) at 1378 cm^−1^ was used for the calibration. The linear dependence of the *A*(*δ*(CH)) with the concentration of oil (Fig. 5, right inset) allowed us to derive the extinction coefficient *ε*_*δ*(CH)_ using Beer–Lambert's law (*ε*_δ(CH)_ = 1259 ± 21 mol^−1^Lcm^−1^). From the experimental identification of *ε* in the δ(CH) domain (*ε*_*δ*(CH)_), the extinction coefficient *ε*_*ν*(CH)_ of the main *ν*(CH) band of oil, centered at 2923 cm^−1^ and corresponding to asymmetric stretching, was determined. Its integrated absorbance wass first fitted using a Voigt function after linear background subtraction between 3000 and 2800 cm^−1^. *ε*_*ν*(CH)_ was then derived from the oil spectrum (Fig. 10) using the relation:

(1) $$\varepsilon_{\nu \left(\mathrm{CH}\right)}=\frac{A\left(\nu \left(\mathrm{CH}\right)\right)\times \varepsilon_{\delta \left(\mathrm{CH}\right)}}{A\left(\delta \left(\mathrm{CH}\right)\right)}$$

The resulting value was *ε*_*ν*(CH)_ = 12, 582 ± 779 mol^−1^Lcm^−1^.

## RESULTS AND DISCUSSION

The results and discussion are presented in three parts, corresponding to the sequential stages of interaction between the oil drops and the banana leaf: the moment of impact of individual drop, the subsequent spray spreading across the surface, and finally, the penetration of the oil into the internal tissues of the leaf.

### Drop impact

**Figure 6:**
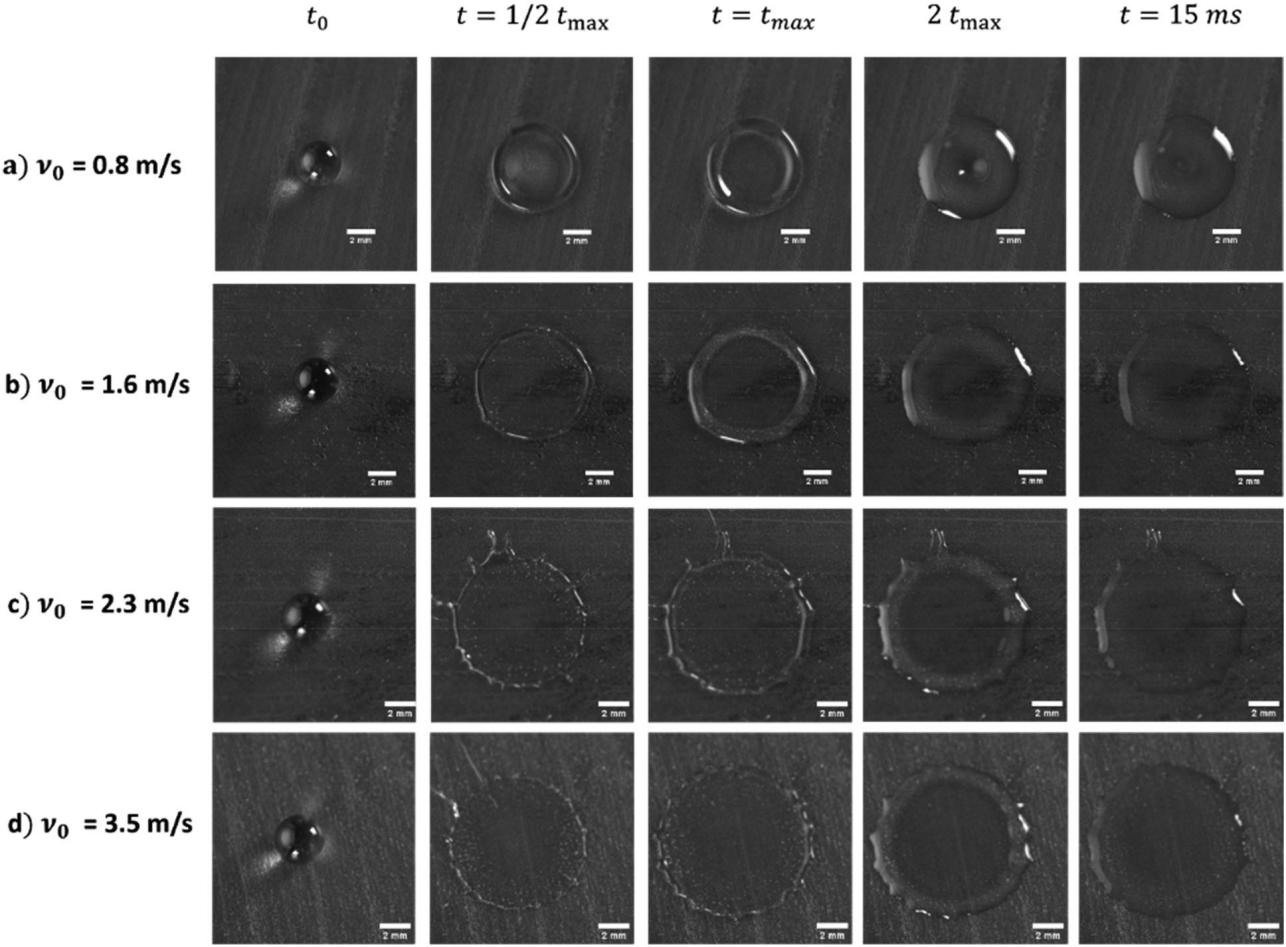
Snapshots taken at successive times during the expansion of a liquid sheet of mineral oil after impact on a banana leaf at different impact velocities *v*_0_; (a) 0.8 m s^−1^, (b) 1.6 m s^−1^, (c) 2.3 m s^−1^ and (d) 3.5 m s^−1^. The impact time is defined as *t*_0_ = *h/v*_0_, and *t*_max_ corresponds to the maximum expansion of the sheet. *t* = 15 ms corresponds to the last point of the plateau (see Fig. 7(a)). The scale bar represents 2 mm.

Figure 6 presents four series of successive snapshots from oil drop impact experiments conducted at various impact velocities *v*_0_. A key observation is the expected increase in the maximum impact parameter *β*_max_ reached by the droplet as the impact velocity increases. Additionally, for all velocities, we observe a monotonic growth of the impact parameter *β*(*t*) over time, until it reaches a plateau value *β*_max_, which depends on the impact velocity, without any droplet retraction.

**Figure 7:**
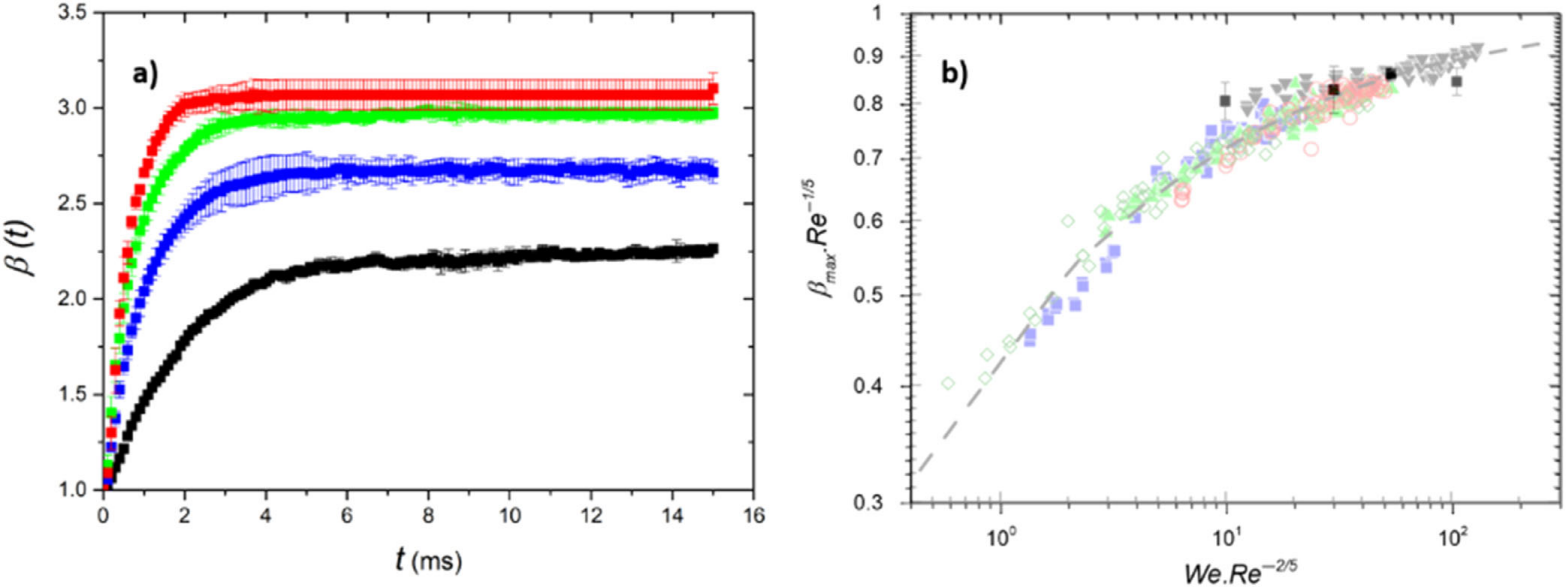
(a) Time evolution of the impact parameter *β(t)* for pure oil droplets at different impact velocities (black: 0.8 m s^−1^, blue: 1.6 m s^−1^, green: 2.3 m s^−1^ and red: 3.5 m s^−1^), and (b) *β*_max_Re^−1/5^ as a function of We Re^−2/5^ for mineral oil (black square markers), compared to the results of Laan *et al* ^23^ obtained on reference flat and wettable surfaces.

Figure 7a shows the time evolution of the impact parameter *β*(t) for various impact velocities ranging from 0.8 to 3.5 m/s. For *v*_*0*_ ≥ 2.3 m/s, we observe a weak splash regime,^21^ where ligaments emerge from the outer edge of the droplet after impact. This phenomenon is typical for low‐viscosity droplets.^22^ Although some ligaments break, forming some secondary droplets, most remain attached to the original droplet. At velocities greater than 3.5 m/s, more ligaments break, leading to an increased number of secondary droplets.

Laan *et al* ^23^ reported the existence of a master curve for the impact of Newtonian fluids on a solid surface, interpolating between the capillary regime and the viscous regime, expressed as βmaxRe−15=fWeRe−25 (see Fig. 7b), where the Weber number $\text{We}=\frac{\mathrm{\rho D}\nu_{0}^{2}}{\gamma }$ compares the effect of inertial forces to capillary forces, and the Reynolds number Re=ρDν0μ. quantifies the ratio of inertial forces to viscous forces. Here, *ρ, μ*, and *γ* denote the density, dynamic viscosity, and surface tension of the liquid, respectively. We verify that our experimental data (black squares in Fig. 7(b))) align reasonably well with the master curve, despite the complexity of the banana leaf's surface roughness. Thus, the impact experiments show that oil droplets spread more widely as impact velocity increases, reaching a stable coverage without retraction. The experimental data confirm predictable spreading dynamics.

Although the drop impact experiments were performed using millimetric oil droplets (D₀ ≈ 3 mm), which are larger than typical spray droplets encountered under field conditions, the underlying impact physics can be discussed in a unified dimensionless framework. Over the entire size range from millimetric to sub‐millimetric droplets, the Bond number Bo = ρgD^2^/γ remains much smaller than unity, indicating that gravity plays a negligible role at impact. As a consequence, droplet dynamics are primarily governed by the Weber number (We), comparing inertia to capillarity, and the Reynolds number (Re), quantifying viscous dissipation. For a completely wetting liquid such as mineral oil on banana leaves, droplets in this size range are therefore expected to belong to the same qualitative impact regime, characterized by inertia–capillarity–viscosity balance, monotonic spreading, and absence of rebound. While the quantitative evolution of the impact parameter β(t) may differ for sub‐millimetric spray droplets, the observed spreading‐dominated regime should remain valid.

These results are directly relevant for phytosanitary applications, as they demonstrate that spray performance can be optimized by adjusting droplet impact velocity to ensure uniform and effective deposition on leaf surfaces.

### Spray spreading

While single‐drop impact experiments provide insight into local spreading dynamics, phytosanitary treatments rely on the collective behavior of multiple droplets deposited as a spray, whose macroscopic spreading emerges from successive impact, spreading, and coalescence events.

**Figure 8:**
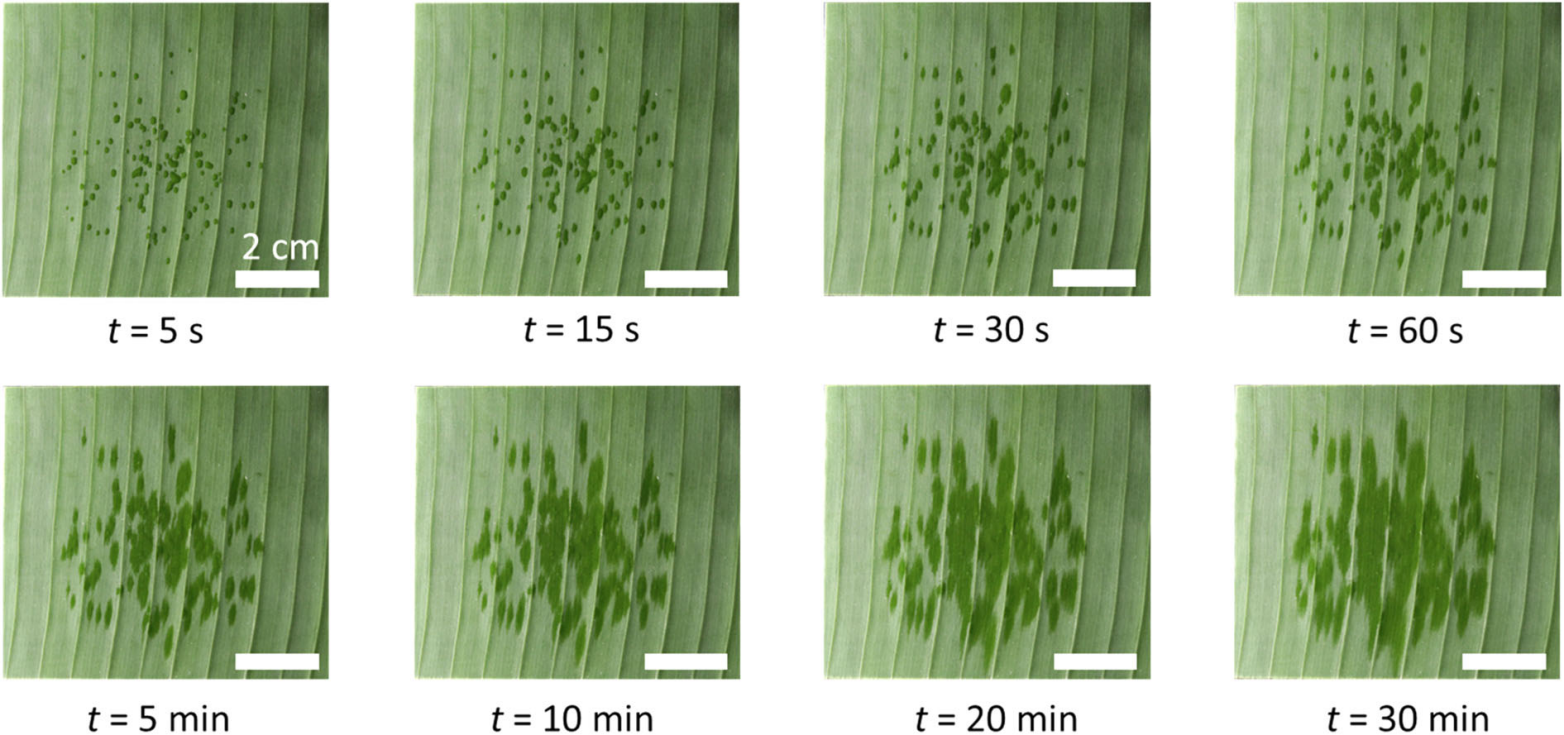
Spreading of mineral oil spray droplets on banana leaf surface over time. Bar = 2 cm.

The spray spreading process is recorded for 35 min. The first image is captured after 5 s, which corresponds to the time required to release the droplet onto the grid and remove the grid from the camera's field of view. We observe that the spreading occurs anisotropically, progressing significantly faster in the direction parallel to the secondary veins. By contrast, spreading in the direction perpendicular to the secondary veins is limited (see Fig. 8).

**Figure 9:**
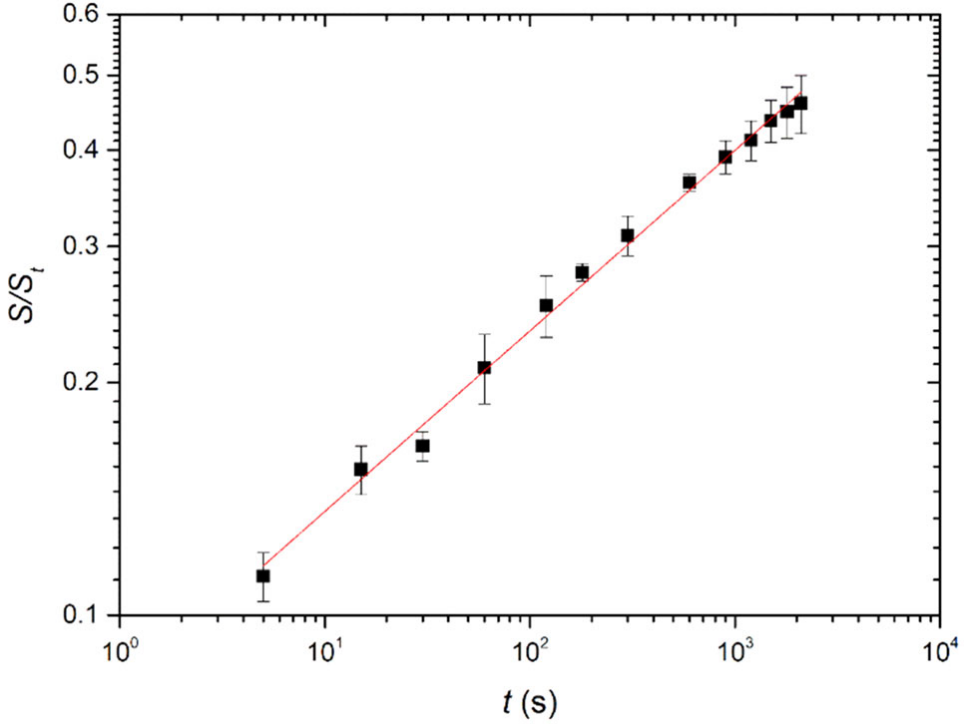
Time evolution of the coverage rate of mineral oil spray droplets on a banana leaf in log–log scale. The red curve represents the fit according to Tanner's law $\left(t\right)\propto t^{0.23\pm 0.02}$, and the error bars indicate the standard deviation across three experiments.

The area of the oil‐covered surface, *S*(*t*), is normalized by the total surface area of the banana leaf, *S*_*t*_, which is 49 cm^2^. We define the coverage rate as *P (t) = S(t)/S*_*t*_. Our observations show that *P(t*)∝ *t* ^0.23±0.02^ (see Fig. 9). This evolution is consistent with Tanner's law^24^ which describes the spreading kinetics of an individual sessile drop on a smooth surface with a radius evolving as *R(t*)∝*t* ^1/10^.

In the case of a spray, the overall spreading process results from the successive spreading of numerous individual droplets and their subsequent coalescence upon contact. Each droplet initially spreads according to the capillary‐driven dynamics described by Tanner's law, and when two droplets merge, the liquid bridge connecting them grows with a power‐law in time whose exponent depends on the same spreading mechanism, as demonstrated by Sellier and Trelluyer.^25^ Thus, if both individual droplet spreading and droplet coalescence obey similar kinetic laws, it is reasonable to consider that the macroscopic spreading of a spray — as a sequence of these microscopic events — follows Tanner‐like dynamics at the global scale.

The anisotropic roughness of the banana leaf, characterized in Section “*Surface roughness*”, likely plays a central role in this directional spreading. The alignment of micro‐ and mesoscopic ridges associated with the secondary veins can promote preferential spreading along the vein direction by locally reducing flow resistance. Such structures may act as open microchannels facilitating capillary‐driven motion of the liquid, a mechanism reminiscent of the open‐channel flows reported by Gerdes *et al*.^26^ Similar guided wetting phenomena on anisotropic substrates have also been discussed.^27^, ^28^ Although such structures may act as preferential pathways for liquid spreading, this interpretation remains speculative, as the present experiments do not directly resolve flow at the scale of individual veins or grooves. Further investigations would be necessary to determine whether this anisotropy arises purely from surface energy gradients or also from channelized transport along the leaf architecture.

We emphasize that this directional spreading ensures better coverage of the surface of the leaf, which are key pathways for pest and pathogen movement. The evolution of the coverage rate following a power law indicates predictable and efficient coalescence of droplets on the leaf surface. Such controlled and homogeneous spreading behavior is advantageous for phytosanitary applications, as it promotes uniform deposition and enhanced protection without excessive runoff or product loss.

### Impregnation

#### Infinite films

**Figure 10:**
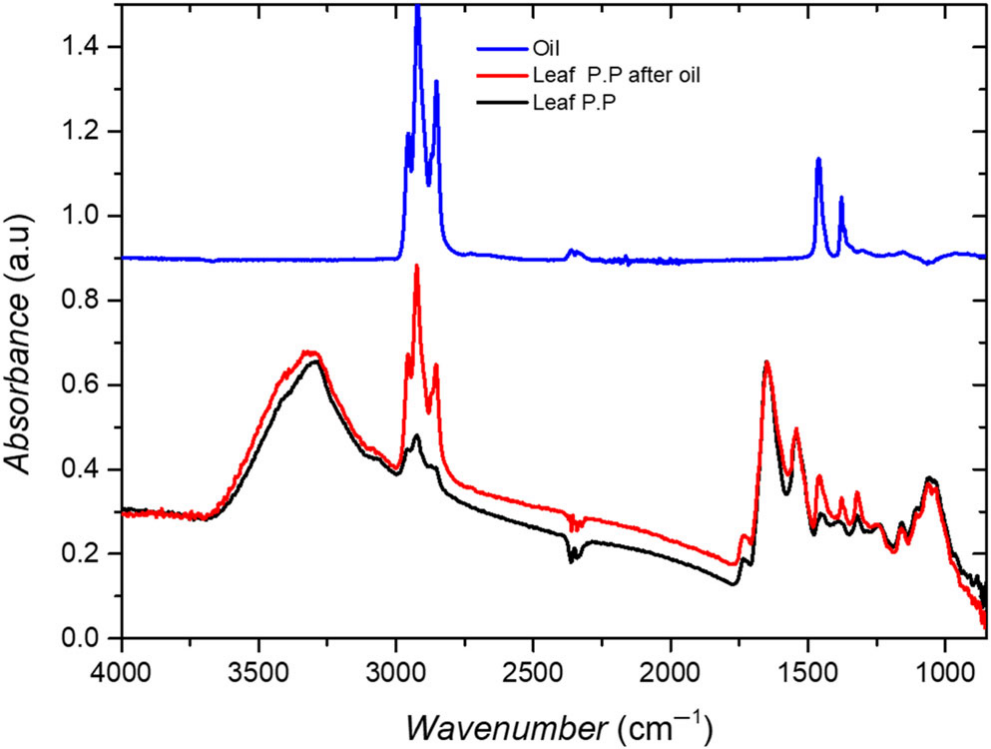
IR spectra of untreated P.P (black), P.P palisade parenchyma after impregnation with mineral oil for 48 h (red), and pure mineral oil (blue).

The IR spectrum of the mineral oil, shown in Fig. 10, reveals two main features: the stretching vibration bands *ν*(CH) between 3000 and 2800 cm^−1^ and the deformation vibration bands *δ*(CH)between 1500 and 1300 cm^−1^.

After 48 h of impregnation with an infinite film of oil on a banana leaf, the IR spectra of the palisade parenchyma (P.P) exhibit a significant oil fingerprint in the *ν*(CH) domain compared to that of an untreated leaf (Fig. 10), suggesting the presence of mineral oil in the P.P after treatment. In the *δ*(CH) domain, the oil signatures overlap with intense and complex features of the P.P, leading to focus our analysis on the *ν*(CH) domain. In other regions of the spectrum, no significant modification of the P.P features is observed, suggesting that the chemical structure of the P.P remains unchanged after impregnation. Chemical mapping in the oil fingerprint domain, specifically the *ν*(CH) band between 3000 and 2800 cm^−1^, is performed before and after impregnation by integrating the absorbance as a function of position across the leaf.

**Figure 11:**
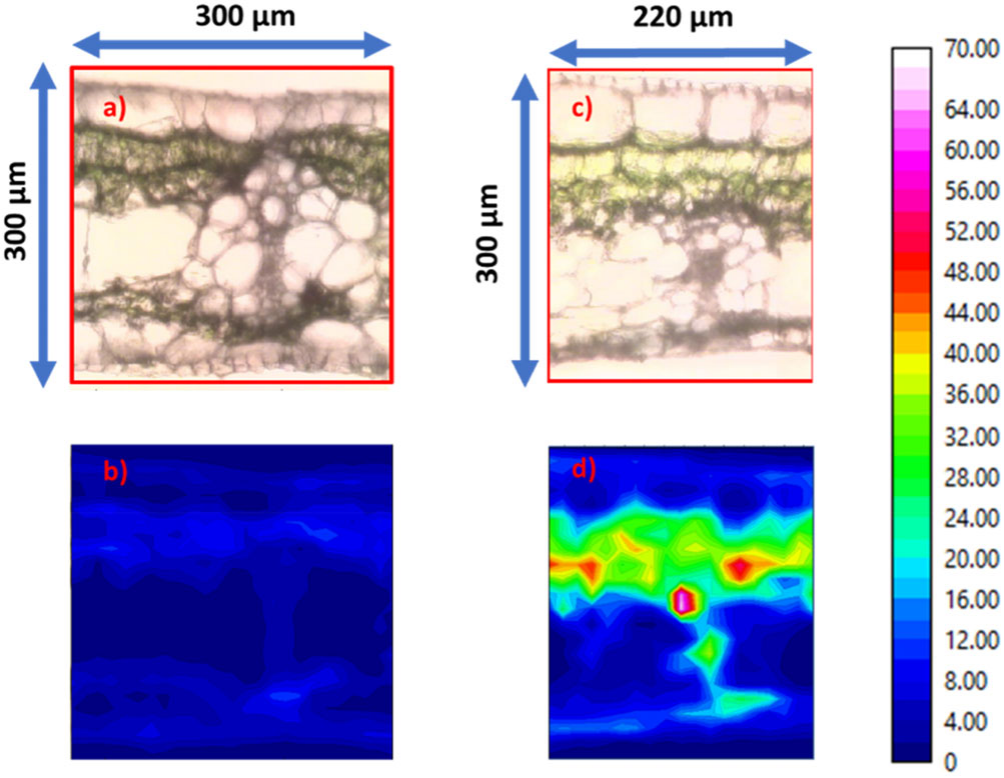
(a) Optical image of a cross‐section of a banana leaf without oil contact, and (b) corresponding chemical mapping in the spectral range of *ν*(CH) (3000–2800 cm^−1^). (c) Optical image of a cross‐section of a banana leaf after 48 h of contact with mineral oil and (d) corresponding chemical mapping in the same spectral range.

**Figure 12:**
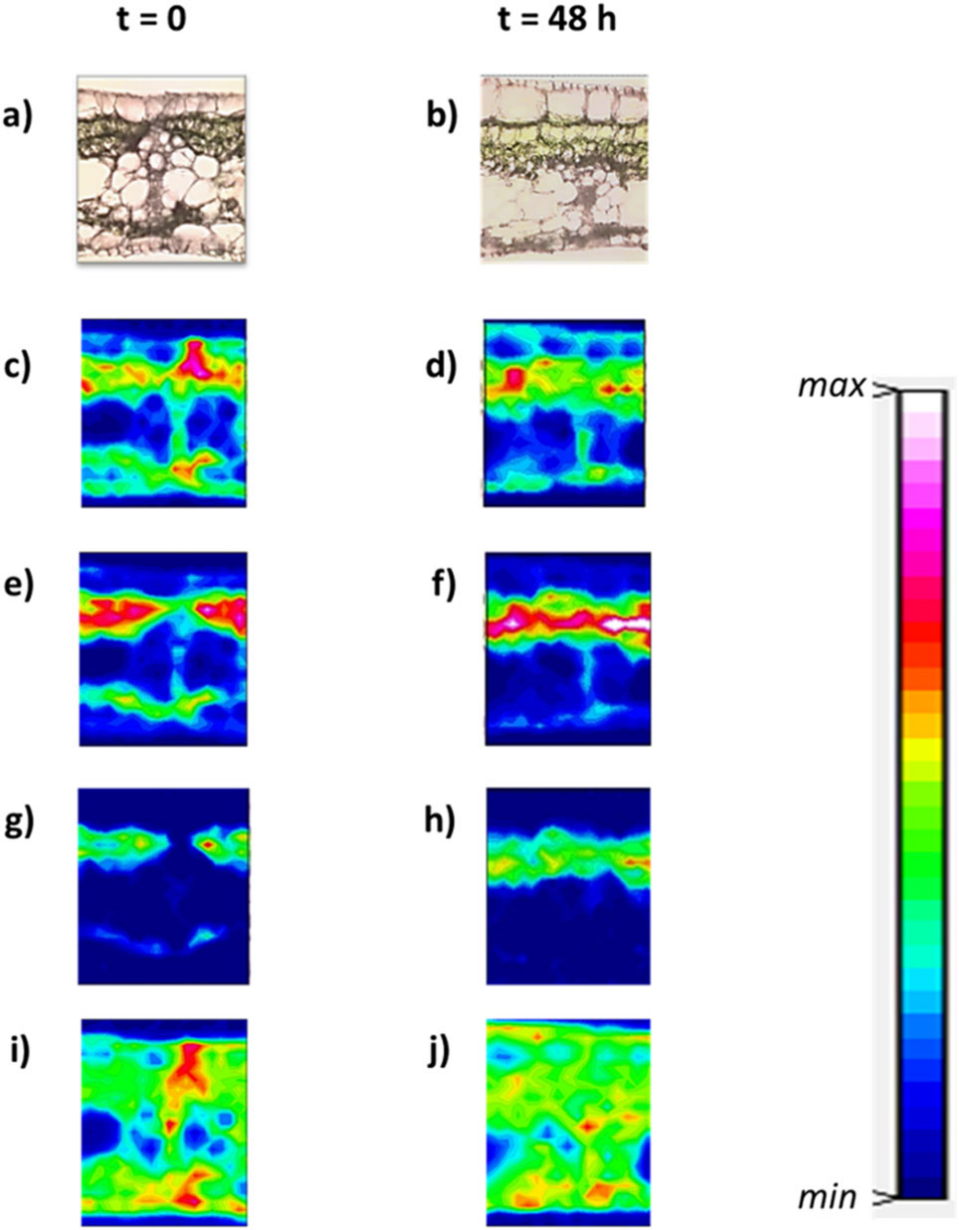
Optical image of a cross‐section of the leaf before oil impregnation (left) and after 48 h of impregnation (right), along with corresponding chemical mappings before and after impregnation. (a) and (b): optical images; (c) and (d): *ν* (OH) and *ν* (NH) (3700–3000 cm^−1^), min 0 a.u., max 160 a.u.; (e) and (f): amide I (1700–1580 cm^−1^), min 0 a.u., max 30 a.u.; (g) and (h): amide II (1580–1480 cm^−1^), min 0 a.u., max 10 a.u.; (i) and (j): polysaccharides (1135–935 cm^−1^), min 0 a.u., max 40 a.u.

Figure 11 compares the optical image of the cross‐section with the corresponding chemical maps of *ν*(CH). The results show that the absorbance across the leaf thickness is significantly altered after impregnation. The P.P, as well as the phloem and xylem, exhibit high intensities compared to the control leaf cross‐section, with values ranging between 35 and 70 a.u. This indicates that the mineral oil penetrates the banana leaf, accumulating mainly in the P.P. Additionally, it migrates to the vascular buy, as expected. Conversely, we observe (see Fig. 12) that the IR mapping specific to the chemical fingerprints of the leaf: *ν*(OH) and *ν*(NH) domain between 3700 and 3000 cm^−1^, amide I domain between 1700 and 1580 cm^−1^, amide II domain between 1580 and 1480 cm^−1^, and polysaccharide vibration domain between 1135 and 935 cm^−1^ remains similar before and after contact. This result demonstrates that the chemical structure of the leaf is not significantly affected by mineral oil impregnation. IR analyses show that the mineral oil efficiently penetrates the banana leaf and accumulates mainly in the palisade parenchyma (P.P.). Despite this impregnation, the overall chemical structure of the P.P. and other leaf tissues remains unchanged.

#### Quantitative analysis of the impregnation kinetics of oil in the palisade parenchyma

To quantify the concentration of mineral oil present in the P.P., the Beer–Lambert law is applied. The concentration C(*x, t*) is derived from the relative absorbance *A*(*t*) − *A*_0_, according to the equation:

(2) $$C\left(x,t\right)=\frac{A\left(t\right)-A_{0}}{\varepsilon_{\nu \left(\mathrm{CH}\right)}l}$$

here *l* is the thickness of the leaf cross‐section (40 μm), *A*_0_ is the absorbance corresponding to the intrinsic *ν*(CH) of the leaf before contact in the 2800–3000 cm^−1^ range, and *A*(*t*) is the absorbance of the leaf's *ν*(CH) after contact within the same spectral range.

The quantification of the integrated molar extinction coefficient, *ε*_*ν*(CH)_ for the *ν*(CH) features is necessary. Significant variations in *ε*_*ν*(CH)_ are observed between 2800 and 3000 cm^−1^. Density functional theory‐based calculations have been performed to simulate the paraffinic oil IR spectrum using the well‐known B3LYP hybrid functional including van der Waals corrections (D3) coupled to the 6‐31G(d,p) basis set.^29^ From these calculations, we determine that the ratio of the molar extinction coefficients for the main asymmetric and symmetric *ν*(CH) stretches is 2, which agrees well with our experimental findings, particularly for the features at 2923 cm^−1^ and 2852 cm^−1^. Consequently, we perform the quantification using the main *ν*(CH) band centered at 2923 cm^−1^, which accounts for 37% of the integrated absorbance of the *ν*(CH) features of the oil in the 2800–3000 cm^−1^ range, as determined from Voigt function fittings. The molar extinction coefficient, *ε*_*ν*(CH)_), for the main *ν*(CH) feature is determined following the procedure described in Section “Micro‐Infrared Spectroscopy”.

**Figure 13:**
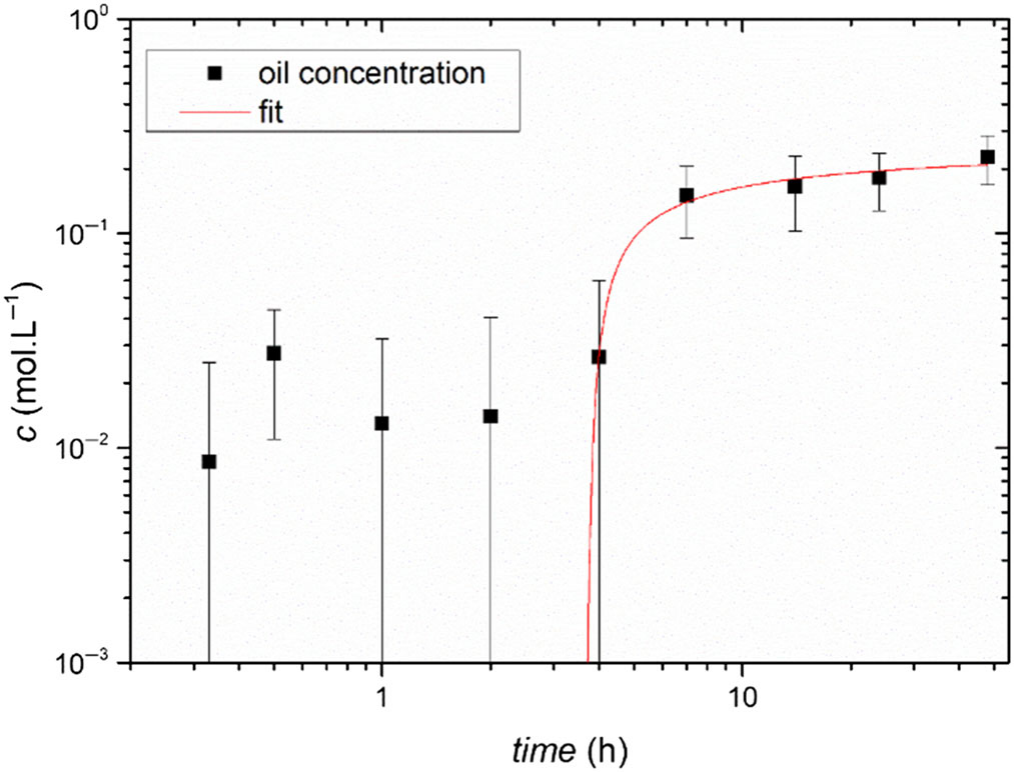
Temporal evolution of oil concentration in the P.P.at depth (*x* = 98.5 μm) corresponding to the middle of the thickness of the parenchyma domain, along with the corresponding fit using Eqn 4. The best fitted parameters are $t_{\mathrm{lat}}=3.6\pm 0.3\;h$ and $D=1.2\pm 0.8\times 10^{-8}{\mathrm{cm}}^{2}s^{-1.}$

The temporal evolution of the concentration of oil impregnated in the P.P. is then studied by converting absorbance into concentration from ten randomly extracted spectra. The concentration is plotted as a function of impregnation time in Fig. 13, with error bars representing the standard deviation of the ten concentration values. Two regimes are clearly identified. Up to a latency time *t*_lat_ = 3.5 h, there is no variation in concentration, which remains zero within the margins of uncertainty. Subsequently, a sublinear increase in concentration over time is observed. This result suggests a diffusive impregnation process preceded by a latency period, which can be modeled by solving the one‐dimensional diffusion equation with an infinite reservoir and an initial latency period:

(3) $$\frac{\mathrm{dC}}{\mathrm{dt}}=-D\frac{d^{2}C}{dx^{2}}$$

with $C\left(x=0,t\right)=C_{0}$ and $C\left(x>0,t-t_{\mathrm{lat}}=0\right)=0$

where *D* is the apparent effective diffusion coefficient of mineral oil in the banana leaf. The solution to this equation is:

(4) $$C\left(x,t\right)=C_{0}\left(1-\mathrm{Erf}\left(\frac{x}{2\sqrt{D\left(t-t_{\mathrm{lat}}\right)}}\right)\;\right)$$

where the error function is defined as: $\mathrm{Erf}\left(y\right)=\frac{2}{\pi }\int_{0}^{y}e^{-t^{2}}\mathrm{dt}$.

The result of the fit using the diffusion model (Eqn 4) of the temporal evolution of oil concentration in the P.P. at depth *x* (*x* = 98.5 μm) corresponding to the middle of the thickness of the P.P. domain is shown Fig. 13. The values of *D* and *t*_lat_ are derived from the fit. The best fitted parameters are $t_{\mathrm{lat}}=3.6\;\pm \;0.3\;h$ and $D=1.2\;\pm \;0.8\;\times \;10^{-8}{\mathrm{cm}}^{2}s^{-1}$. One can notice that each experimental point corresponds to an average value obtained for fourteen different positions in the P.P. for one leaf sample. We have also verified the reproducibility of the results performing mapping on three different leaves sample. The effective diffusion coefficient of oil in banana leaves is comparable to that found for a lipid molecule in a lipid membrane.^30^ Although there is no direct physical interpretation of the latency time, − most likely corresponding to a barrier effect of the cuticle. Such a latency is consistent with the cuticle acting as a lipid diffusion barrier for hydrophobic molecules, as documented in the literature^31^

It is interesting to note that the common agricultural practice is to repeat an oil‐based phytosanitary treatment if it has rained less than five hours before the first spraying, in agreement with the measured value.

Thus, we show that oil penetration in banana leaves follows a diffusion process and provides an effective diffusion coefficient. The diffusion model accurately describes the temporal evolution of oil concentration highlighting the cuticle's barrier effect and explains why repeated phytosanitary treatments are typically spaced several hours apart.

#### Sprays

**Figure 14:**
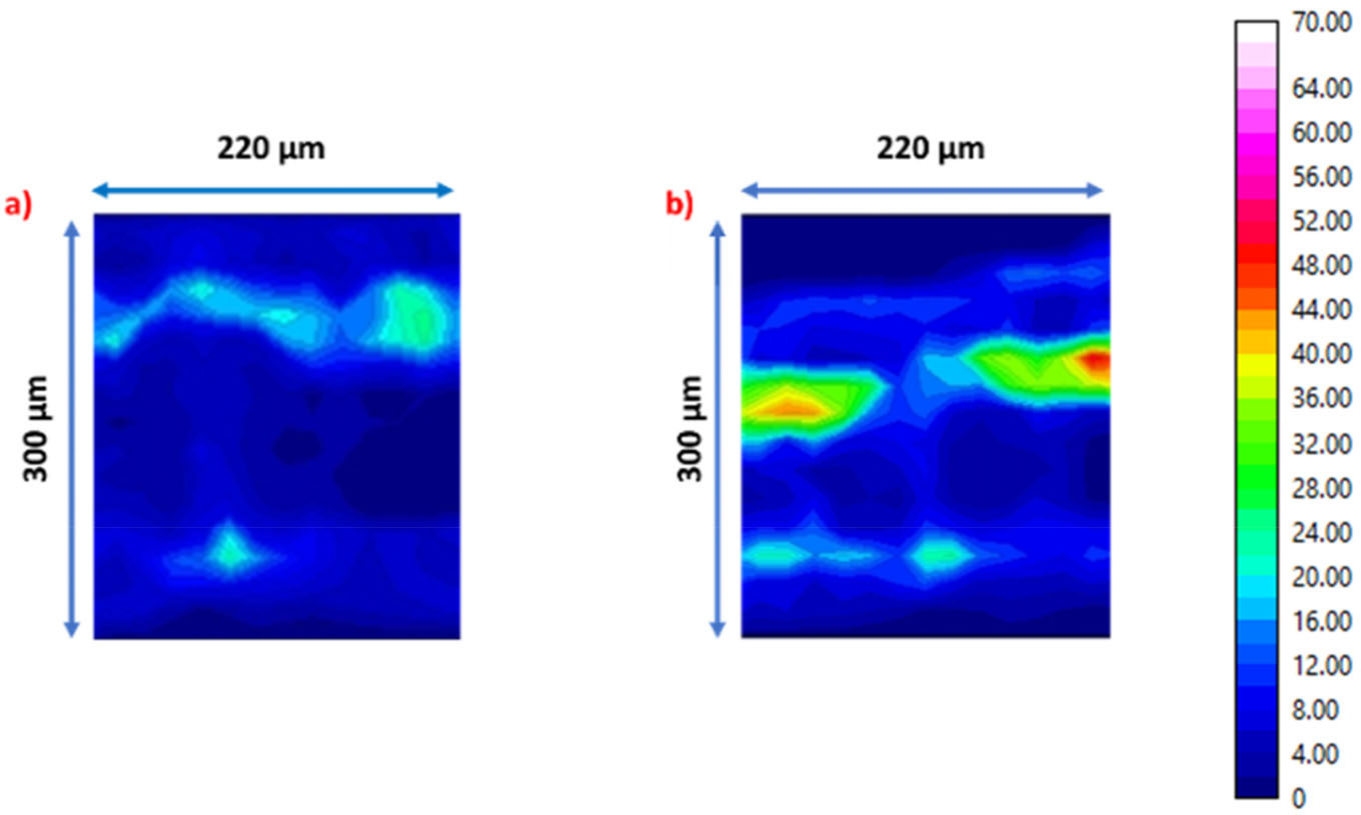
(a) Chemical mapping of the cross‐section in the spectral range of *ν*(CH) without oil contact, and (b) chemical mapping of the cross‐section 24 h after oil spraying in the same spectral range

After 24 h of contact with an oil spray on the leaf (see Section “Spray spreading”), corresponding to real field spreading conditions, 40 μm‐thick cross‐sections are prepared using the previously described procedure. The infrared mapping of the *ν*(CH) band reveals the presence of oil in the P.P. after spray impregnation (see Fig. 14), as previously observed for an infinite oil film. Notably, the intensities of the ν(CH) features are similar in the P.P., but no impregnation is observed in the xylem and phloem. In contrast to infinite‐film impregnation, spray application leads to a more localized oil distribution, with the palisade parenchyma remaining the dominant compartment in the IR maps. This difference is plausibly related to the much smaller delivered oil volume, shorter effective contact time, and the heterogeneous nature of spray deposition compared with an infinite reservoir. This result confirms the efficiency of oil impregnation by spraying.

## CONCLUSIONS AND PERSPECTIVES

This study offers a novel physicochemical and multi‐scale characterization of the interaction between mineral oil and banana leaves—covering, for the first time, the full sequence of droplet impact, spray spreading, and tissue impregnation. By combining high‐speed imaging, image analysis, and micro‐infrared spectroscopy, we provide quantitative insights into each stage of the process under conditions representative of real‐world phytosanitary treatments.

Our results reveal that oil droplets exhibit impact dynamics characterized by rapid spreading without rebound and minimal splash generation, even at high velocities. Spreading on the banana leaf surface is strongly anisotropic, guided by the leaf's multiscale structural organization, particularly the orientation of secondary veins. Remarkably, spray coverage follows Tanner's law—originally derived for single droplet spreading—highlighting an unexpected analogy between macroscopic spray behavior and individual droplet dynamics.

Most notably, we demonstrate that mineral oil does not merely coat the leaf surface but slowly diffuses into the palisade parenchyma, following a Fickian diffusion process preceded by a measurable latency time (~3.6 h). This delay aligns with empirical agronomic practices recommending reapplication if rain occurs shortly after spraying. We also provide, for the first time, a quantitative measurement of the oil diffusion coefficient within banana tissue.

Furthermore, previous studies by Cavalcante *et al* ^32^ have shown that, following spore germination on the banana leaf surface, the mycelium of Mycosphaerella fijiensis penetrates through the stomata and spreads within the internal tissues, where the plant activates defense responses—particularly within the foliar parenchyma. In this context, our demonstration that mineral oil not only adheres to the surface but also penetrates the inner leaf tissues, with preferential accumulation in the palisade parenchyma, is of particular interest. This suggests that mineral oils may exert their protective effect not solely through surface action, but also by interacting with the inner tissues targeted during early fungal invasion, potentially contributing to enhanced protection or priming of local defense responses.

## CONFLICT OF INTEREST

The authors declare no conflict of interest.

## AUTHOR CONTRIBUTIONS

C.L. and J.‐L.B. conceived and supervised the project. A.A. led the experimental investigation, with assistance from S.B. for the micro‐IR experiments. P. H. performed the *ab initio* calculations of the IR oil spectra. C.L., J.‐L.B., and A.A. performed the analysis and co‐wrote the manuscript, with the help of J.‐.L. V. J.‐L.V and F.G. took charge of the optical microscopy measurements and the preparation of microtome sections.

## Data Availability

The data that support the findings of this study are available on request from the corresponding author. The data are not publicly available due to privacy or ethical restrictions.
